# Advances in lipid-based nanocarriers for breast cancer metastasis treatment

**DOI:** 10.3389/fmedt.2022.893056

**Published:** 2022-08-18

**Authors:** Ingrid Joun, Sheri Nixdorf, Wei Deng

**Affiliations:** ^1^School of Chemical Engineering, Faculty of Engineering, University of New South Wales, Sydney, NSW, Australia; ^2^Graduate School of Biomedical Engineering, Faculty of Engineering, University of New South Wales, Sydney, NSW, Australia; ^3^School of Biomedical Engineering, Faculty of Engineering and IT, University of Technology Sydney, Ultimo, NSW, Australia

**Keywords:** breast cancer metastasis, liposomes, targeting strategy, nanomaterial-based drug delivery, lipid nanoparticles (LNPs)

## Abstract

Breast cancer (BC) is the most common cancer affecting women worldwide, with over 2 million women diagnosed every year, and close to 8 million women currently alive following a diagnosis of BC in the last 5-years. The side effects such as chemodrug toxicity to healthy tissues and drug resistance severely affect the quality of life of BC patients. To overcome these limitations, many efforts have been made to develop nanomaterial-based drug delivery systems. Among these nanocarriers, lipid-based delivery platforms represented one of the most successful candidates for cancer therapy, improving the safety profile and therapeutic efficacy of encapsulated drugs. In this review we will mainly discuss and summarize the recent advances in such delivery systems for BC metastasis treatment, with a particular focus on targeting the common metastatic sites in bone, brain and lung. We will also provide our perspectives on lipid-based nanocarrier development for future clinical translation.

## Overview of breast cancer and its metastasis

Breast cancer (BC) has become the most prevalent cancer in the world with over 2 million women diagnosed every year, and close to 8 million women currently alive following a diagnosis of BC in the last 5-years (1, 2). Together, Australia and New Zealand have the highest incidence of BC globally with over 20,000 new cases annually (1, 3). Our understanding of the mechanisms of tumor development and progression have significantly improved over the last 50 years and have led to better BC screening, diagnostic and treatment modalities, resulting in a 91.5% 5-year survival rate post-diagnosis (3, 4). Even so, BC remains one of the top causes of cancer-associated mortality and resulted in 685,000 deaths worldwide in 2020 alone (1). Ninety percent of these deaths are attributed to metastatic disease, either present at diagnosis (6–7%) or due to disease progression (15–30%) (5–7). The 5-year overall survival of advanced BC is around 25% but varies depending on the metastatic site, the most common being bone (75–80% cases; 22.8–43.4% 5-year survival), lung (60–70% cases; 16.8% 5-year survival), liver (50% cases; 8.5% 5-year survival) and brain (15–30% cases; 2–25-month survival) (8–12). It is apparent from these figures that we must focus our research efforts on treating metastatic tumors if we wish to further improve BC survival outcomes.

Through the rise of genetic characterization of cancers, it has been found that an ever-evolving heterogeneity exists between and within individual tumors. Different BC subtypes have been identified and correlate with metastatic potential and overall survival, with Triple Negative Breast Cancer (TNBC) having the worst prognosis due to the inefficiency of Estrogen Receptor (ER), Progesterone Receptor (PR) and Human Epidermal Growth Factor Receptor 2 (HER2) targeted therapies (13). Recent analyses have further confirmed that tumors continue to evolve as they invade the body, with more extensive genetic alterations found in metastatic sites when compared to primary tumors (14).

Bone metastases were found to develop predominantly from Hormone Receptor (HR)+/HER2- tumors, whereas 15–27% of liver, lung and brain metastases originated from poorly differentiated tumors with a triple negative profile (15). Genetic screening of metastatic BCs by Bertucci et al. (14) identified 9 driver genes in the HR+/HER2- subtype with *TP53, RB1* and *NF1* mutations resulting in therapy resistance and poorer overall survival. As expected TNBC metastases displayed additional biallelic loss of HR-associated genes (14). In general, metastatic BCs were downregulated in the genes responsible for cellular differentiation (GATA3) and hormonal response (BCL2), and upregulated for those involved with cell cycle progression, DNA repair and survival (e.g., CCNB1, FGFR4) (16). Additionally, disease progression involved dysregulation of numerous signaling pathways such as the Hedgehog, Notch, TGF-β and Wnt pathways, which all play a role in cancer cell migration and invasion through activation of the epithelial to mesenchymal transition process (17). With a plethora of knowledge continually being collected on the molecular mechanisms of metastatic BC, there is great potential for the development of targeted therapies, with many currently undergoing the clinical trial phase of testing to determine their efficacy as single or combination agents in BC therapy (12, 18).

Current best practice for treatment of BC involves a combination of surgery, radiotherapy, chemotherapy, endocrine and targeted therapies, with the extent and combination of treatment dependent on the stage, grade, histological and genetic characteristics of the tumor(s) (19–21). The ER, PR, HER2 and Ki67 biomarkers are routinely used to guide treatment, specifically in the case of metastatic disease (see Table 1).

**Table 1:** Treatment options for metastatic BC (19, 78, 79).

**Biomarker profile**	**Therapy**
ER+/PR+/HER2-	Endocrine therapy (e.g., Tamoxifen) + single-agent chemotherapy, ± palbocyclib/letrozole/fulvestrant
HER2+	Taxane + trastuzumab + pertuzumab; Trastuzumab-emantine, lapitanib, trastuzumab ±single-agent chemotherapeutics
ER-/PR-/HER2-	Chemotherapy: Anthracyclines and/or taxanes; Doxorubicin + cyclophosphamide; Epirubicin + cyclophosphamide May use: Docetaxel + cyclophosphamide; Cyclophosphamide/methotrexate/5-fluorouracil
PD-L1+	Nab-paclitaxel + atezolizumab

While personalized treatment approaches have improved BC survival, delivery of effective doses is often associated with higher non-target toxicity (22, 23). The use of nanoparticle drug carrier systems to treat metastatic BC tumors has the potential to target multiple metastatic sites simultaneously, incorporate combination therapies, and use higher drug concentrations without the cytotoxic effects associated with systemic delivery on non-encapsulated therapies (24). In this review, we will mainly focus on examining the efficacy of currently available liposomes and lipid nanoparticles (LNP) and discussing their therapeutic potential for the treatment of metastatic BC.

## Basics of liposomes and LNP

Liposomes and LNP have been extensively used as delivery vehicles for the treatment of cancer, genetic disease, and other health issues, making them an essential asset in the pharmaceutical industry (25–28). While they have slight differences in composition and structure, these lipid-based nanoformulations generally encapsulate a variety of pharmaceutical cargos within a protective, outer layer of lipids. Liposomes are bilayer or multilayer lipid vesicles in which an aqueous volume is entirely enclosed by a membrane composed of amphiphilic phospholipids (29–32). They are composed of phospholipid molecules which are able to self-assemble into a lipid bilayer or multilayer vesicles in an aqueous environment (33). During this process, the liposomes can selectively load therapeutic agents by entrapping hydrophilic molecules in the aqueous core and hydrophobic molecules in the lipid bilayer (34). LNP consist of a solid lipid core, manufactured from either solid lipids or a mixture of liquid and solid lipids (35). Such structures were especially engineered toward encapsulating nucleic acids (such as RNA and DNA), therefore LNP are the most popular non-viral gene delivery system (36). Liposomes and LNP drug delivery systems have many advantages over systemic drug delivery (37). They can prevent unwanted degradation of drug and reduce exposure to healthy cells (38). This in turn increases biodistribution and payload of drug at target sites (39). Moreover, these systems are less toxic compared with other nanoparticles due to their biocompatible and bio-degradable characteristics (40, 41).

Both liposomes and LNP can be engineered to enter cancer cells *via* a passive or active targeting strategy (42). Passive targeting relies on the enhanced permeability and retention (EPR) effect which means well-vascularised tumors exhibit discontinuous endothelial cell lining (43). This leaky endothelium can be used as an access point by nanoparticles smaller than 150 nm in size (44). Once liposomes/LNP penetrate the tumor, they are usually internalized by cells and enter into the interior structures of the cell such as the lysosomes and cytoplasm (45, 46). However, when using liposomes/LNP to target cancer metastases *via* this strategy the major challenge was the fact that early metastatic sites lack leaky endothelium, resulting in limited cellular internalization of nanoparticles (47, 48). Hence, active targeting strategies could be used to overcome this problem due to its capability to enhance nanoparticles' binding to cancer cells (48). Active targeting incorporates targeting molecules such as antigens, antibodies, enzymes, and ligands to the surface of liposomes/LNP. These targeting molecules have high affinity interaction with the biomarkers overexpressed by the tumor cells, leading to enhanced accumulation of liposomes/LNP within the tumor cells (49, 50).

## Metastatic breast cancer treatments *via* liposomes and LNP

Despite medical advances having remarkably changed the management of BC patients over the past decades, metastases still pose a significant challenge due to their resistance to therapeutic agents, molecular heterogeneity, and the presence of physiological barriers at various organ sites (24, 51). The vastly distributed nature of metastasis also means systemic chemotherapy treatment does not take into consideration the immense differences in tumor microenvironments (52, 53). With the development of liposome/LNP technology, these nanoformulations exhibit clear advantages, including enhanced drug properties and pharmacokinetics as well as reduced drug toxicity. In addition, they can be tailored to simultaneously target cancer cells and the tumor microenvironment for enhanced targeting and treatment capabilities. Various anticancer drugs have been tested in liposomal/LNP formulations to improve their efficacy such as Doxorubicin (DOX), Paclitaxel (PTX), zoledronic acid (ZOL), Mitomycin C (MMC) and siRNA (24, 36, 54, 55). We mainly analyzed recent studies on the metastatic BC treatments *via* these nanoformulations (Table 2). These reports demonstrated the improved therapeutic efficacy at the cellular level and in an animal model by engineering liposomes/LNP based on the EPR effect and active targeting strategy. Table 2 summarizes the liposomes/LNP delivery systems with various ligands for BC metastasis targeting and treatment.

**Table 2:** Recent studies of BC metastasis treatment using liposomes/LNP.

**Site**	**Delivery system and cargos**	**Nanocarrier target mechanism**	***In vitro* study**	***In vivo* study**	**References**
Bone	Liposomes containing ZOL	Asn-Gly-Arg (NGR) can recognize aminopeptidase N (APN) expressed in tumor endothelial cells	N/A	The therapeutic effect of different ZOL formulations were assessed in tumor bearing mice	(54)
Bone	Liposomes containing DOX	Asp8 is used to target the bone structure by chelating calcium ions on the surface of hydroxyapatite; Folate targets the folate receptors overexpressed on cancer cells	Folate density on cellular uptake, Asp8 on hydroxyapatite binding and cell cytotoxicity were examined	Therapeutic efficacy of DOX liposomal formulations on pain behavior and NIR fluorescence imaging on liposome biodistribution	(56)
Bone	Liposomes containing PTX	Glu6 is a novel glutamic oligopeptide that can target the bone structure through ionic interaction with Ca^2+^; RGD peptide targets the α_v_β_3_ integrin overexpressed in bone metastatic cells and osteoclasts	The binding capability of Glu6-RGD modified liposomes to hydroxyapatite (HAP), a promising target for selective drug delivery to bone, was assessed	Therapeutic efficacy of liposome-formulated PTX was assessed in Balb/c nude mice bearing MDA-MB-231 tumors	(57)
Brain	Terpolymer-lipid NP containing DOX & MMC	PS 80 present in the terpolymer LNP is used to engage apolipoprotein E (ApoE) in blood circulation to extravasate the brain microvessels; iRGD is used to target the αvβ3 and αvβ5 integrins overexpressed in TNBC	Cellular uptake and cytotoxicity of liposome formulation on MDA-MB-231 cell line and RAW 264.7 macrophages	Mouse models intracranially injected with MDA-MB-231 cells were used to observe biodistribution of DOX, and host survival using various LNP formulation	(58)
Brain	Liposomes containing irinotecan	EPR effect	N/A	Survival rate/brain tumor progression of mice, drug exposure of irinotecan and active metabolite SN-38 were investigated by using DiI5-liposomal irinotecan	(59)
Brain	PTX- and TWF1 siRNA-loaded liposomes	BRBP1 peptide is used to enhance cellular uptake in TNBC brain cancer cells	Cellular uptake and cytotoxicity were examined for targeted liposomes	Anti-tumor activity and targeting capability of PTX/siRNA liposome formulations in a mouse model with brain metastasis were examined	(60)
Brain	Liposomes containing PTX	Folic acid and dNP2 peptide can synergistically enhance penetrability of the BBB and tumor targeting capability	*In vitro* BBB model by using 3D tumor spheroids of 4T1 cells; penetrability of liposomes was assessed on this model	*In vivo* imaging, anti- tumor activity and survival were examined using brain tumor mice models	(61)
Lung	DOX & MMC co-loaded polymer lipid NP	Conjugation of RGD to target the αvβ3 integrin overexpressed in lung cancer cells	*In vitro* cytotoxicity of nanoparticles with different RGD ligand densities were investigated	Biodistribution studies, survival rate and dose tolerance were examined on metastatic lung tumor mice	(55)
Lung	Polymer- lipid NP (PLN) containing NCTD	Conjugation of RGD is used to recognize ITGA5 overexpressed in TNBC cells and lung metastasis	Cellular uptake activities and *in vitro* inhibitory effects of PLN and RGD-PLN were assessed	PLN's long-term accumulation, biodistribution and tumor growth control in a mouse model bearing lung tumor were investigated	(62)
Lung	Liposome containing DOX	ICAM1- and EGFR-neutralizing antibodies are used as they are overexpressed in TNBC cells with low expression in normal healthy cells	Cell cytotoxicity and cellular uptake of these liposomes in MDA-MB-231 cells were examined	Lung metastasis model in nude mice were used to test liposomes' treatment capability	(63)
Lung	Liposome containing DOX	EGFR and RGD dual targeting strategy	N/A	*In vivo* tumor targeting and treatment capabilities of DOX RGD/EGFR-liposomes in a mouse model with lung metastasis were assessed	(53)
Lung	DOX Liposomes	EPR effect	N/A	Liposomes loaded with ICG and Dox were injected into BALB/c mice to evaluate biodistribution in primary tumor and lung metastasis, compared with free drug	(44)

### Lung metastasis targeting and treatment

Lung metastasis is a major cause of death in BC patients (64). To overcome off-target effect of traditional chemotherapy, targeted liposomes were engineered to deliver therapeutics directly to cancer cells and endothelial cells of the tumor vasculature. One typical ligand that can aid in targeting BC lung metastasis is the peptide Cilengitide, an Arg-Gly-Asp (RGD) mimetic which targets the αvβ3 integrin (65). These integrins are overexpressed in BC cells and angiogenic endothelium and play a role in inducing metastatic tumor growth. RGD peptide has been extensively explored in various liposome formulations to enhance their cancer cell targeting capability in BC and its lung metastasis, due to its high serum stability, low immunogenicity, and easy synthesis protocol (66, 67). Many studies demonstrate its ability for tumor-associated extravasation and deep tissue penetration (66, 68).

**Figure 1:**
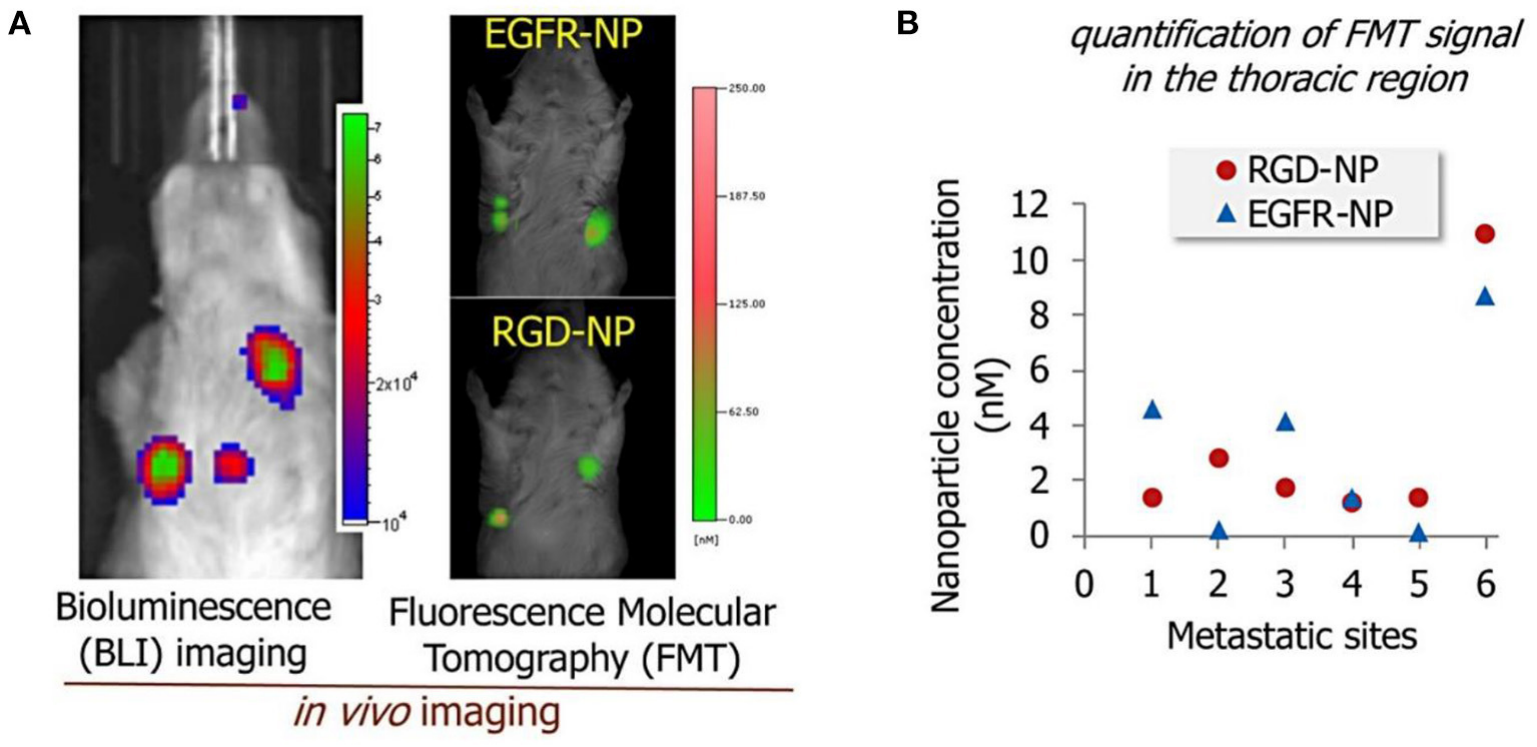
**(A)** Bioluminescence images of the lung metastasis development (left) and florescence signals from EGFR-modified and RGD-modified liposomes in the metastatic sites (right). **(B)** The response of cancer metastasis in a mouse model to treatment by using bioluminescence imaging signal. The signal in the thoracic region is shown for the treatment at days 3, 4, and 5. The treatments included non-targeted NP (NT-NP), RGD-NP, EGFR-NP, dual-ligand NP, and free DOX (n = 6–8 mice per treatment). All nanoparticle formulations were administered at 7.5 mg/kg DOX (two-way ANOVA with repeated measures), adapted from (53).

One recent work reported by Covarrubias et al. (53) used RGD and EGFR antibodies to target EGFR and αvβ3 integrin overexpressed by BC cells. Multi-ligand targeted liposomes demonstrated an enhanced sensitivity to expression patterns of certain targetable receptors at various stages of lung metastasis. The authors found these two single-ligand formulations had different targeting performance varying from one metastatic site to the next (Figure 1A). They also claimed that the dual-ligand targeting liposomes exhibited better accumulation performance in the metastatic lung tumor regions by 2-fold compared to single targeting liposomes (RGD-NP and EGFR-NP). Therapeutic efficacy of targeted and non-targeted liposomes was then compared. The group of mice treated with the dual-ligand targeting liposomes (dual-ligand NP) displayed lower bioluminescence signals compared with other treatment conditions (Figure 1B). However, this study only considered treatment at the very early stages where the micro metastasis would be extremely difficult to diagnose in clinical settings.

**Figure 2:**
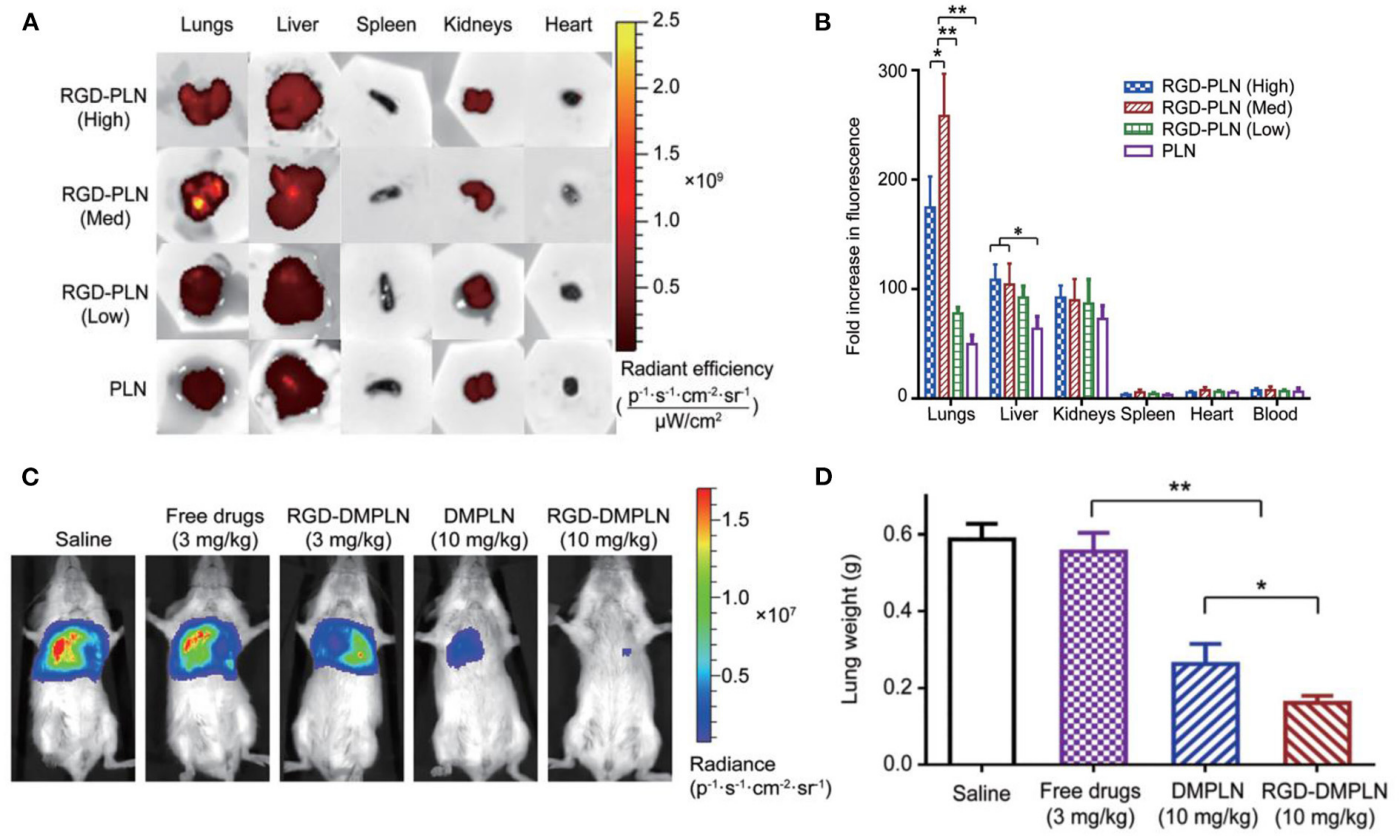
**(A)** Qualitative presentation of organ biodistribution at 4 h. **(B)** Quantitative presentation of ex vivo organ biodistribution at 4 h. The data are represented as mean ± SD, n = 3, *P < 0.05, **P < 0.01. **(C)** Representative *in vivo* bioluminescent images of mice from each treatment group on day 28 after tumor inoculation. **(D)** Quantification of metastases by lung metastasis area index (n = 9 for each group; *P < 0.05, **P < 0.01), adapted from (55).

Another study reported by Zhang et al. (55) demonstrated the therapeutic effect of polymer-lipid nanoparticles (PLN) modified with RGD on the lung metastasis growth of BC. The PLN were coated with RGD at different concentrations from 1.7 μmol/L (1%, low), 16.6 μmol/L (10%, medium) to 49.7 μmol/L (30%, high). MDA-MB-231 cancer cells exhibited minimum viability when treated with PLN modified with RGD and loaded with DOX and MMC, compared with non-targeted PLN and free drugs. The maximal accumulation in the metastatic lung tumor was observed when using RGD-PLN (10%, medium), compared to nanoparticles with either low or high RGD concentration (Figures 2A,B). The RGD-PLN displayed significant lung tumor inhibiting capability in a mouse model bearing MDA-MB-231-luc-D3H2LN cells, with a 31-fold decrease in tumor bioluminescence radiance compared with free drugs (Figure 2C). This formulation also lowered the lung metastasis area index by 4.0-fold, compared with pure drugs (Figure 2D).

### Brain metastasis targeting and treatment

BC brain metastasis (BCBM) is a major cause of morbidity of BC patients since many currently available therapies are unable to cross the blood brain barrier (BBB). The BBB is a physiological and morphological barrier that resists most compounds from crossing from the blood to the brain, maintaining the homeostasis of the brain microenvironment (69, 70). Therefore, the BBB provides an obstacle to the entry of drugs and other exogenous compounds into the central nervous system. By contrast, the blood brain tumor barrier (BBTB) is a leaky structure caused by the brain tumor's damage to the BBB's function (69). By taking advantage of this leaky structure, we can engineer liposomes/LNP to overcome the BBTB and enter the brain tumor *via* a passive and active targeting strategy.

Mohammad et al. (59) developed liposomes incorporating irinotecan (nal-IRI) to target and treat BCBM *via* the EPR effect. Liposomes were intravenously injected into a mouse model bearing human brain seeking BC cells (MDA-MB-231Br-Luc). The authors found that these liposomes not only crossed the BBTB, but also localized within the cancer cells. In addition to preferential accumulation at the tumor site, the nal-IRI formulation exhibited better tumor control capability compared with pure irinotecan. Liposomal irinotecan-treated groups significantly prolonged median survival to 50 days in the 50 mg/kg treatment group and 48 days in the 10 mg/kg group. However, treatment with conventional irinotecan (50 mg/kg) did not show improvement in survival (35 days).

**Figure 3:**
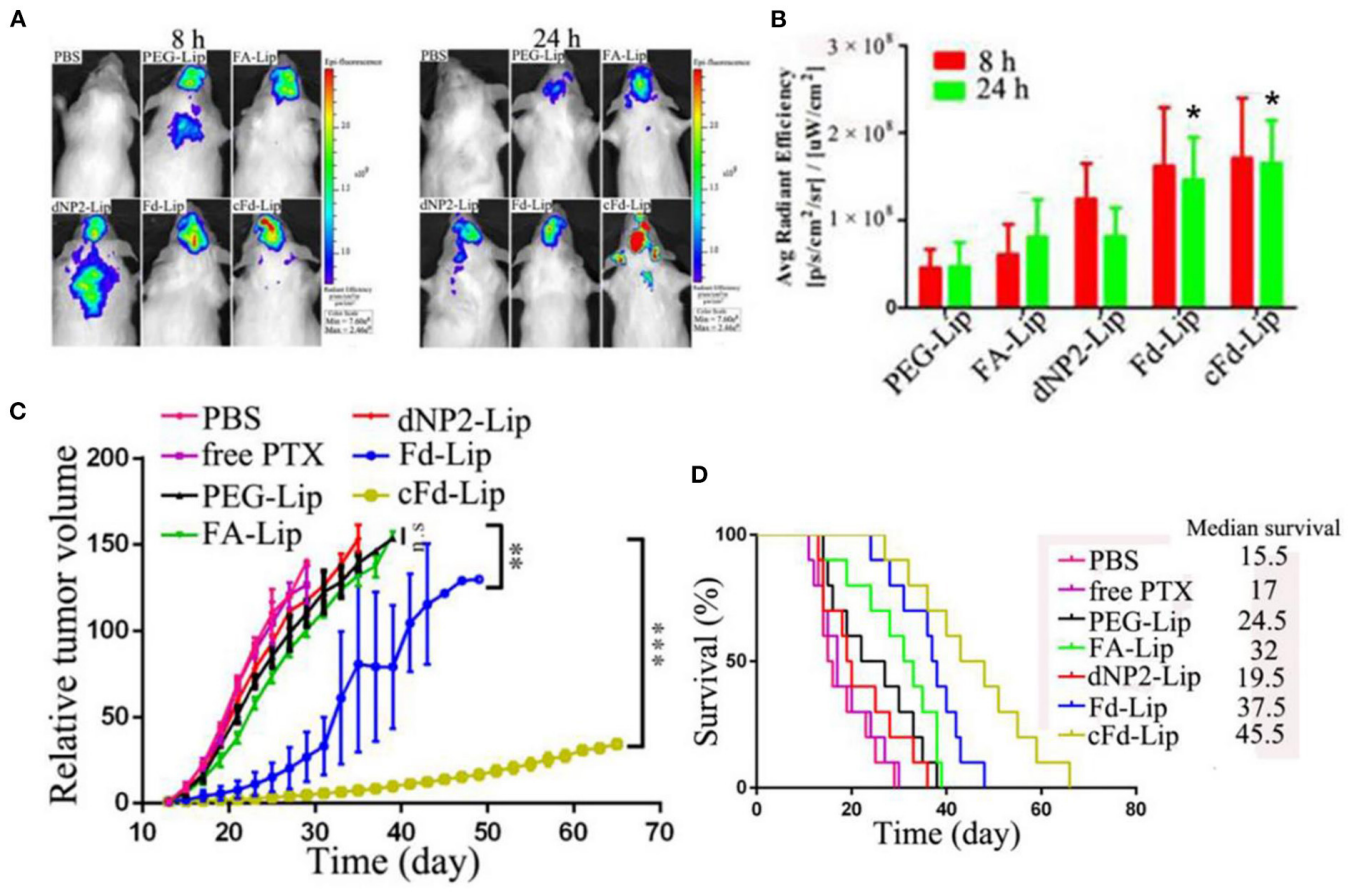
**(A)** The *in vivo* imaging of brain metastases of 4T1 breast cancer-bearing mice after iv injection of liposomes at 8 and 24 h. **(B)** The average fluorescence intensity of ex vivo brain tumors of mice after injection of liposomes at 8 and 24 h (mean ± SD, n = 3, *P < 0.05 compared with PEG-Lip, FA-Lip and dNP2-Lip groups). **(C)** The relative tumor volume after treatment with various liposome formulations (mean ± SD, n = 10, **P < 0.01, ***P < 0.005). **(D)** Kaplan–Meier survival curves of mice treated with liposome formulations (n = 10), adapted from (61).

Other recent work reported dual targeting liposomes for BCBM treatment (61). These liposomes were modified with dNP2 and folic acid (FA) to enhance their targeting capability on cancer cells. dNP2 is a BBB permeable peptide which can increase BBB transmigration/cellular uptake; FA can specifically bind to folate receptors (FR) overexpressed on the surface of various cancer cells. An *in vitro* 3D BBB model was used to test the penetrating performance of these liposomes. The acid-cleavable FA and dNP2 dual targeting liposomes (cFd-Lip) was superior to non-cleavable dual targeting liposomes (Fd-Lip) and single ligand modified liposomes at pH 6.8 in promoting the cellular uptake and deep penetration in *in vitro* models. The non-cleavable FA-modified liposomes exhibited lower internalization which suggests steric hinderance of the FA on the surface of liposomes. *In vivo* therapeutic efficacy of these liposomes was tested in a BALB/c mouse model bearing 4T1 cells. Dual targeting liposomes exhibited higher accumulation in brain compared with other liposome formulations (Figures 3A,B). This phenomenon could be attributed to the synergistic BBB targeting and penetration of FA and dNP2. Additionally, tumor growth was significantly inhibited and survival rate of tumor-bearing mice was prolonged after the treatment with such liposomes (Figures 3C,D). Despite the compelling anti-tumor effects, these *in vitro* and *in vivo* models may not fully simulate the tumor microenvironment and complexity of the metastatic process.

### Bone metastasis targeting and treatment

Treatment options for BC bone metastasis is hindered by the complex bone microstructure and a lack of an EPR effect due to low blood flow (56). Hence, non-targeted liposomes/LNP may have limited accumulation in the bones due to their size and low penetrability of the bone (71). Active targeting strategies could be taken into consideration when using liposomes/LNP to treat bone metastasis, including targeting both the tumor microenvironment and cancer cells simultaneously.

Ke et al. (56) developed aspartate (Asp8) and folate co-modified liposomes (A/F-LS) incorporating DOX to treat bone metastasis and reduce pain behavior in a mouse model. Asp8 exhibits a negative charge due to the carboxylate ligands, which can chelate calcium ions on the surface of hydroxyapatite (HA), a mineral found only in the bone. Hence Asp8 can target the bone structure. Furthermore, folate is used as a second ligand to specifically target FRs overexpressed on the surface of cancer cells. *In vitro* results demonstrated that the dual-ligand targeting liposomes significantly reduced the MDA-MB-231 cell viability (*p* < 0.5) compared to non-targeted liposomes and free DOX. Moreover, the cellular uptake activities of targeted liposomes were enhanced with Asp8/lipid molar ratio being increased.

**Figure 4:**
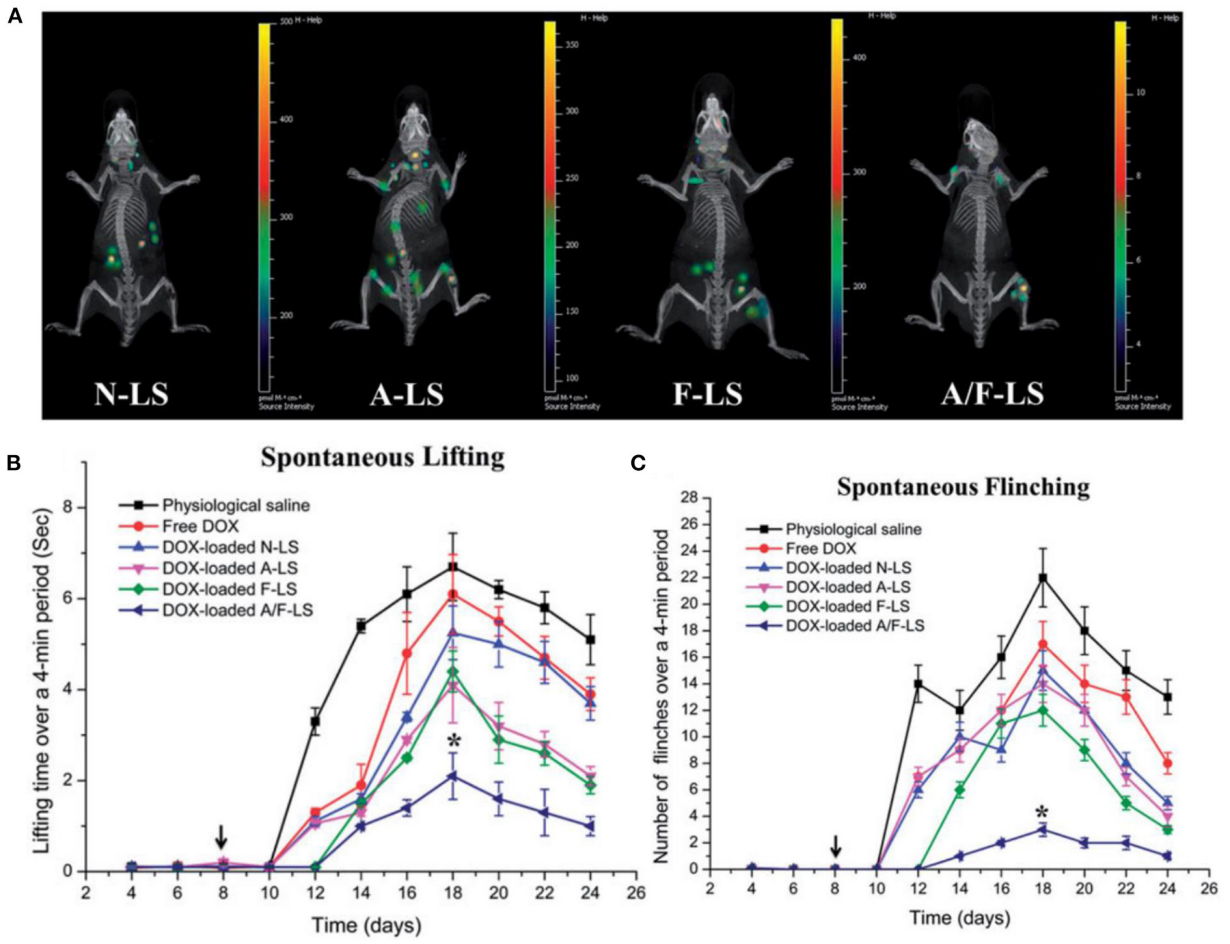
**(A)** Biodistribution of Cy5.5 contained in various liposomes in mice bearing MDA-MB-231 tumors determined by an IVIS^®^ Spectrum-CT. **(B)** Spontaneous time lifting of tumor-bearing right hind limb over a 4-min observation period. **(C)** Number of flinches of tumor-bearing right hind limb over a 4-min observation period, *P < 0.05, adapted from (56).

The *in vivo* study further displayed the bone targeting capability of the A/F-LS and other liposome formulations. Asp8 modified liposomes (A-LS and A/F-LS) exhibited good bone-targeting ability *in vivo* (Figure 4A). More importantly, the accumulation of A/F-LS in the tumor sites in the bone is higher than that of A-LS (Figure 4A), indicating that Asp8 helped with bone-targeting of liposomes with the folate further enhancing their accumulation within the tumors. The authors also evaluated bone pain behavior in MDA-MB-231- bearing mice after the treatments. They found that A/F-LS loaded with Dox can significantly attenuate flinching and lifting time compared with other treatment conditions (Figures 4B,C). These findings indicated that DOX loaded targeted liposomes may enhance therapeutic efficacy in treating bone metastasis including pain relief and overall survival improvement.

Another similar study reported by Zhao et al. (57) demonstrated the bone targeting performance of the liposomes co-modified with glutamic oligopeptides and RGD peptide (Glu6-RGD-Lip). Negatively charged glutamic oligopeptides have high bone affinity through ionic interaction with Ca^2+^ in the mineral component of bone. RGD peptide can help specifically recognize cancer cells. *In vitro* results demonstrated that PTX-loaded liposomes exhibited sustained release behaviors (60% drug released after 48 h incubation in PBS), compared to rapid release of free PTX (80% of drug released within 12 h). Further cellular toxicity assessment demonstrated a 60% reduction in MDA-MB-231 cell viability upon exposure to the Glu6-RGD-Lip. The *in vivo* results revealed that Glu6-RGD-Lip have a higher accumulation of PTX in the bone lesion compared to other liposome formulations. PTX concentration in metastatic bones was 5–8- fold higher using Glu6/RGD-Lip than free PTX and 3–5- fold higher compared to non-targeted liposomes. However, this work did not claim any *in vivo* therapeutic efficacy of such liposomes.

Despite the active targeting strategy leading to higher cellular uptake activities of nanoparticles, compared with the passively targeted counterparts in these studies, the overall therapeutic efficacy of actively targeted nanoparticles vs. passively targeted systems for drug delivery still remains unclear. In addition, physiological challenges such as tumor penetration, relative hypoxia and endosomal escape further limited the therapeutic benefit of actively targeted nanoparticles (72). In order to enhance the efficacy by taking advantage of actively targeted nanoparticles, several considerations should be systematically addressed, such as how to overcome the physiological barriers, control intracellular trafficking of nanoparticles once inside the cell, and achieve intracellular recognition (73–75).

## Conclusion and perspectives

Research efforts into more effective treatment strategies for metastatic BC patients have shown great progress with the use of liposomes and LNP. The emerging formulations with dual targeting ligands, synergistic drugs, and the tunability of physiochemical properties of such nanoparticles have shown clear improvements for drug delivery at the metastatic tumor site. There is tremendous opportunity in this field to engineer such nanoparticles for better cancer targeting and treatment outcomes as well as a better understanding of the metastatic microenvironment. Research is still in its infancy and reproducibility of such complex liposome formulations is mostly unknown (24, 76). These formulations still suffer from poor characterization in relation to specific practices for standardized risk assessments and evaluation of nanotoxicities (76, 77). Although, this is less challenging for passive targeting due to its simpler design (69). In addition, clinical translation hurdles, manufacturing/ scale-up and anatomical barriers presented by these novel strategies need to be considered when discussing the viability of such treatment options. Inherently, with more intricate formulations the efficacy of these liposomes/LNP must outweigh the substantial increase in costs and manufacturing complexities. However, we envision that these challenges would be overcome by the close collaboration between the experts in all relevant fields including liposome/LNP design, industry manufacturing, and safety and efficacy evaluation in *in vivo* settings.

Overall, liposomes and LNP delivery systems display incredible potential in BC metastasis treatment. Studies explored in this review indicate their capabilities on enhanced cancer cell targeting and treatment over systemic chemotherapy. Further research efforts would open more opportunities for clinical translation of such nanomedicines.

## Data availability statement

The original contributions presented in the study are included in the article, further inquiries can be directed to the corresponding author.

## Author contributions

WD led the intellectual content and revised the whole manuscript. IJ drafted the liposome/LNP and treatment section. SN drafted the overview of breast cancer part. All authors edited the manuscript. All authors contributed to the article and approved the submitted version.

## Funding

This work was financially supported by funding (GNT1181889) from the Australian National Health and Medical Research Council and a fellowship award (2019/CDF1013) from the Cancer Institute NSW, Australia.

## Conflict of interest

The authors declare that the research was conducted in the absence of any commercial or financial relationships that could be construed as a potential conflict of interest.

## Publisher's note

All claims expressed in this article are solely those of the authors and do not necessarily represent those of their affiliated organizations, or those of the publisher, the editors and the reviewers. Any product that may be evaluated in this article, or claim that may be made by its manufacturer, is not guaranteed or endorsed by the publisher.
